# Flame-Retardant GF-PSB/DOPO-POSS Composite with Low Dk/Df and High Thermal Stability for High-Frequency Copper Clad Applications

**DOI:** 10.3390/polym16040544

**Published:** 2024-02-17

**Authors:** Ke Zheng, Yizhi Zhang, Jiaxiang Qiu, Guanqun Xie, Zengbiao Huang, Wei Lin, Zhimeng Liu, Qianfa Liu, Xiaoxia Wang

**Affiliations:** 1 Sub Center of Dongguan University of Technology of National Engineering Research Center of Electronic Circuits Base Materials, School of Materials Science and Engineering, Dongguan University of Technology, Dongguan 523808, China; zhengke@dgut.edu.cn (K.Z.); yz_zhang1295@163.com (Y.Z.); liuzm@dgut.edu.cn (Z.L.); 2 School of Environment and Civil Engineering, Dongguan University of Technology, Dongguan 523808, China; qiujx1016@126.com; 3 National Engineering Research Center of Electronic Circuits Base Materials, SHENGYI Technology Co., Ltd., Dongguan 523808, China; huangzb@syst.com.cn (Z.H.); linw@syst.com.cn (W.L.); liuqf@syst.com.cn (Q.L.)

**Keywords:** polymer composites, low dielectric loss, flame retardant, thermal stability, high-frequency copper clad laminate

## Abstract

In the field of high-frequency communications devices, there is an urgent need to develop high-performance copper clad laminates (CCLs) with low dielectric loss (Df) plus good flame retardancy and thermal stability. The hydrocarbon resin styrene-butadiene block copolymer (PSB) was modified with the flame-retardant 9,10-dihydro-9-oxa-10-phosphaphenanthrene-10-oxide/polyhedral oligomeric silsesquioxanes (DOPO-POSS) to meet the demands of high-frequency and high-speed applications. The resulting DOPO-POSS-modified PSB was used as the resin matrix along with other additives to fabricate PSB/DOPO-POSS laminates. At a high-frequency of 10 GHz, the laminates containing 20 wt.% of DOPO-POSS and with a thickness of 0.09 mm exhibited a Df of 0.00328, which is much lower compared with the commercial PSB/PX-200 composite (Df: 0.00498) and the PSB without flame retardancy (Df: 0.00453). Afterwards, glass fiber cloth (GF) was used as a reinforcing material to manufacture GF-PSB/DOPO-POSS composite laminates with a thickness of 0.25 mm. The flame retardancy of GF-PSB/DOPO-POSS composite laminate reached vertical burning (UL-94) V-1 grade, and GF-PSB/DOPO-POSS exhibited higher thermal and dynamic mechanical properties than GF-PSB/PX-200. The results of a limited oxygen index (LOI) and self-extinguishing time tests also demonstrated the superior flame-retardant performance of DOPO-POSS compared with PX-200. The investigation indicates that GF-PSB/DOPO-POSS composite laminates have significant potential for use in fabricating a printed circuit board (PCB) for high-frequency and high-speed applications.

## 1. Introduction

**Figure 1:**
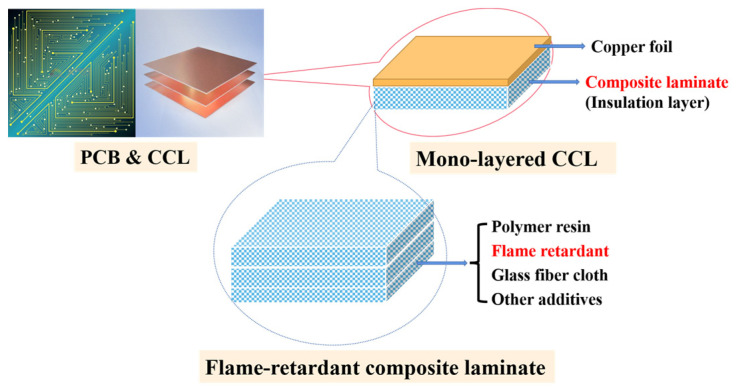
The structure and components of a CCL, a base material of PCB.

The expansion of 5G technologies to various fields [1], including daily consumption [2], healthcare [3], and manufacturing [4], has resulted in an urgent demand for high-performance printed circuit boards (PCBs) for high-frequency information transmission electronics. Copper clad laminates (CCLs) serve as the base material for PCB (Figure 1). Therefore, the development and production of high-frequency and high-speed CCLs is crucial [5,6]. To develop high-performance CCLs, the dielectric loss (Df) and dielectric constant (Dk), two of the most important parameters, must be considered [7]. The Df is the ratio of the energy that flows into a nearby medium (lost) to the energy that travels along the signal path, while the dielectric constant (Dk) represents the insulation capacity. However, as the frequency increases, the Df significantly increases, while the Dk decreases, which is unfavorable for signal transmission [8]. Therefore, it is highly desirable for CCLs to have a low Df and stable Dk at high speeds and frequencies.

CCL consists mainly of copper foil and composite laminate as the insulation layer, which is made from polymer resin, a flame retardant, glass fiber cloth (GF), and other additives (Figure 1) [9]. The performance of high-frequency CCLs is mainly determined by the polymer resin [10]. Polyphenolic and epoxy resins are commonly used for traditional CCLs’ processing. However, the above resins are limited in super-high-frequency environments due to the poor dielectric properties [11]. Hydrocarbon resins with low polarity and low hygroscopicity, such as 1,2-polybutadiene (1,2-PB) [12], styrene-ethylene-butylene-styrene copolymer (SEBS) [13], and styrene-butadiene-styrene copolymer (SBS) [14], are well-known low-dielectric materials. However, flame-retardant modification is essential for the practical application of these polymer resins in industry due to their high flammability [15].

As is well known, various flame retardants have been developed for the flame-retardant modification of resins. In particular, bromo-containing organic compounds have been routinely used, which could effectively reduce the possibility of fire risks and/or slow down the rate of combustion of polymer materials. However, brominated flame retardants tend to release toxic gases or soot in the event of a fire and do harm to both the environment and human health [16]. With increasing concerns about the ecological environment, halogen-free retardants have increasingly drawn the attention of researchers and phosphorous-, nitrogen-, or silicon-containing flame-retardants have emerged in recent years [17,18,19,20]. Among the various halogen-free flame-retardants developed in recent decades, phosphorus-containing compounds are very attractive due to the merits of high flame-retarding performance and low toxicity [21,22,23,24].

As for the flame-retardant modification of hydrocarbon resins, some attempts have been made using phosphorus-containing compounds. However, these compounds may deteriorate the Df of the resin matrix due to their high polarizability and the incompatibility of flame retardants [25]. After conducting a literature survey, it was found that there are few reports on the successful flame-retardant modification of hydrocarbon resins for use in high-performance CCLs. Therefore, the development of satisfactory flame retardants presents a challenge. In addition, high-frequency environments place higher thermal-resistance requirements on electrical insulation materials used for power modules [26]. It is highly desirable to develop an efficient and thermally stable flame retardant for the modification of hydrocarbon resins and at the same time maintain low Dk and Df.

The past decade has witnessed the rapid development of nanotechnology, and a variety of new nano-materials emerged, which opened a new way of developing flame-retardant hydrocarbon resins. Among various kinds of nano-materials, polyhedral oligomeric silsesquioxanes (POSS) has a unique structure with a cubic silica–oxygen skeleton at its core and tunable organic pendant groups (-R) at its shells [27,28]. The weakly polarized group and porous cage-like structure enable POSS to possess low Dk and Df. Zhou et al. [29] discovered that increasing the cage volume of POSS could make the Dk values exhibit a linear decrease, but it had no significant effect on the other properties. The results suggested that POSS had great potential to be used as a modifier to decrease the Dk and Df of CCLs, ensuring high-frequency and high-speed information transmission in communication. Furthermore, the flame-retardant performance of the POSS could be improved by introducing tunable side groups such as P-containing, N-containing, B-containing, or aromatic groups [30,31,32,33,34,35]. 

DOPO-POSS is a kind of modified POSS containing P and Si elements. With high contents of phosphorus and silicon (P, 10.49 wt.%; Si, 9.51 wt.%), DOPO-POSS exhibits the flame-retardant effect of the phosphorus-silicon synergism and has been proved effective in the flame-retardancy of epoxy resins and polycarbonate [36,37,38,39]. The limited oxygen index (LOI) value of DOPO-POSS (5 wt.%) modified TGDDM/DDS (tetraglycidyl diamino diphenyl methane/4,4′-diaminodiphenylsulphone) resins was reported to reach as high as 34.5%, which was obviously higher than the pure resin without DOPO-POSS (29.3%) [40]. And, the DOPO-POSS modified resin was reported to reach the UL-94 V-0 grade with a blowing-out effect. In addition, the cage-like highly symmetrical siloxane structure also endows DOPO-POSS with excellent heat resistance and mechanical properties. Considering its potential as a functional filler for electronic packaging, DOPO-POSS was utilized to modify hydrocarbon resin in order to create new dielectric materials.

To make the DOPO-POSS readily available, our group recently developed a greener synthetic route [41] with better yield and thermal stability as compared with the previously reported method [42]. Here, DOPO-POSS was adopted as a modifier for the preparation of GF-reinforced PSB-composite laminate. Hydrocarbon resin PSB was elaborately selected because of its ultra-low Df (<0.003) at high frequencies. Compared with the corresponding composite laminate modified with a commercial phosphorus flame retardant (PX-200), the as-prepared composite laminate GF-PSB/DOPO-POSS has excellent advantages in its dielectric and thermal properties, showing great potential for use in high-frequency PCBs.

## 2. Materials and Methods

### 2.1. Materials

Styrene-butadiene block copolymer (PSB, 70 wt.% vinyl, 17–27 wt.% styrene; type R100) was purchased from Cray Valley Chemical Co., Ltd. (Guangzhou, China); tetrakis (2, 6-dimethylphenyl)-1, 3-phenylene bis(phosphate) (PX-200) was purchased from Daihachi Chemical Industry Co. Ltd. (Osaka, Japan); polyhedral oligomeric silsesquioxanes-containing phosphorus (DOPO-POSS) was synthesized from a perfect T_8_ cage [41]. All the other chemicals were purchased from Energy Chemical (Shanghai, China), and used as received.

### 2.2. Preparation of DOPO-POSS [41]

In a N_2_ atmosphere, OV-POSS (24.00 g, 37.91 mmol), DOPO (73.76 g, 341.21 mmol), and AIBN (1.25 g, 7.58 mmol) were dissolved in toluene (110 mL) in a 500 mL flask. The mixture was stirred at 80 °C for 10 h and then cooled down to r.t. The up-layer toluene was decanted and the residual paste was dissolved in CH_2_Cl_2_ (90 mL). The resulting solution was added in ethyl acetate (1200 mL) under stirring to precipitate a white powder. Filtration and drying at 100 °C under a vacuum overnight afforded 72.8 g of DOPO-POSS (81% yield).

### 2.3. Preparation of Composite Laminates

**Figure 2:**
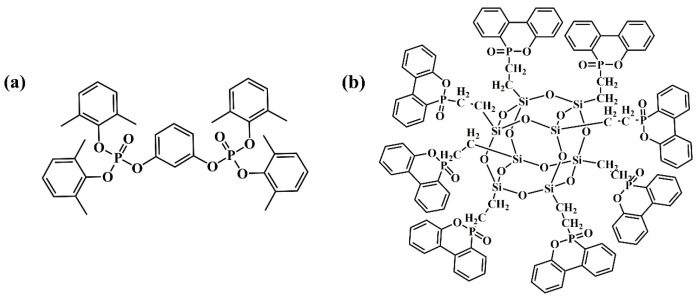
The molecular structures of (**a**) PX-200 and (**b**) DOPO-POSS.

The formulations for the preparation of PSB composites or GF-reinforced PSB (GF-PSB) composite laminates are listed in Table 1. For the preparation of PSB composites, PSB resin and other additives including SiO_2_ filler and a curing agent were added in toluene with certain percentage contents (~65 wt.% solid content) in the presence or absence of the flame retardant (PX-200 or DOPO-POSS, Figure 2). The resulting mixture was stirred for 4 h to produce a varnish, which was then applied to a release film, dried in an oven, and solidified into a ~0.09 mm thickness of a dielectric layer and used as a sample for Dk and Df tests.

For the preparation of GF-PSB composite laminates, the varnish was prepared as above. The GF was immersed into the varnish for 5 min before it was taken out to pass through a roller. Then, the cloth was cut into the required sizes and dried for 12 h at 120 °C. Finally, several layers of the prepregs were stacked together and subjected to hot pressing at 200 °C for 150 min to afford the composite samples with a thickness of 0.25 mm.

### 2.4. Characterization Methods

#### 2.4.1. IR Analysis

Attenuated total reflection Fourier transformed infrared spectroscopy (ATR-FTIR) was performed on a Nicolet iS10 spectrometer (Thermo, Waltham, MA, USA) in the range of 400–4000 cm^−1^.

#### 2.4.2. Dielectric Properties Analysis

The microwave dielectric properties were measured using a N5230A vector network analyzer (Agilent, Santa Clara, CA, USA) combined with a split post-dielectric-resonators (SPDR) method. The dimensions of the composite laminate were 80 × 80 × 0.80 mm^3^. The Dk and Df values of the samples at 10 GHz were recorded. 

#### 2.4.3. Thermogravimetric Analysis

Thermogravimetric analysis (TGA) measurements were performed with a Q500 instrument (TA, New Castle, USA) under a nitrogen atmosphere, with the samples being heated from 50 to 800 °C at a heating rate of 10 °C/min and a gas flow rate of 60 mL/min.

#### 2.4.4. LOI Analysis

The LOI values were measured on a FTT0077 oxygen index meter (FTT, Britain, Derby, UK) with bar dimensions of 130 × 6.5 × 3.2 mm^3^ according to ASTM D2863-2008.

#### 2.4.5. Vertical Burning Analysis

Vertical burning (UL-94) tests were conducted using a CZF-2 instrument (Jiangning Analysis Instrument Factory, Nanjing, China) with the dimension of 125 × 13 × 3.2 mm^3^ according to ASTM D3801. Each group of experiments was performed three times in parallel, and the results were the average of the tests. After the test, the burning grade and the sum of self-extinguishing time (*t*_1_ + *t*_2_) were recorded.

#### 2.4.6. Cone Calorimeter Analysis

Cone calorimeter (CC) tests were performed on a FTT0007 cone calorimeter (FTT, Britain, Derby, UK) according to the ISO 5660 standard. The samples with a size of 100 × 100 × 4 mm^3^ were placed on a holder and irradiated at a heat flux of 50 kW/m^2^.

#### 2.4.7. SEM Analysis

Scanning electron microscopy (SEM) experiments were performed with a JSM-6701F scanning electron microscope (Shimadzu, Tokyo, Japan), and the samples were prepared using low-temperature fracturing, and gold was spurted on the surface.

#### 2.4.8. Dynamic Mechanical Analysis

A dynamic mechanical analysis (DMA) was carried out with a DMA Q800 V213 Build 96 dynamic mechanical analyzer (TA, New Castle, USA). The dimensions of the specimens were 20 × 12 × 0.9 mm^3^ for the storage modulus and glass transition temperature (*T*_g_) detection. The measuring frequency was 1 Hz. The temperature was varied with a heating rate of 3 °C/min.

## 3. Results and Discussion

### 3.1. ATR-FTIR Characterization

**Figure 3:**
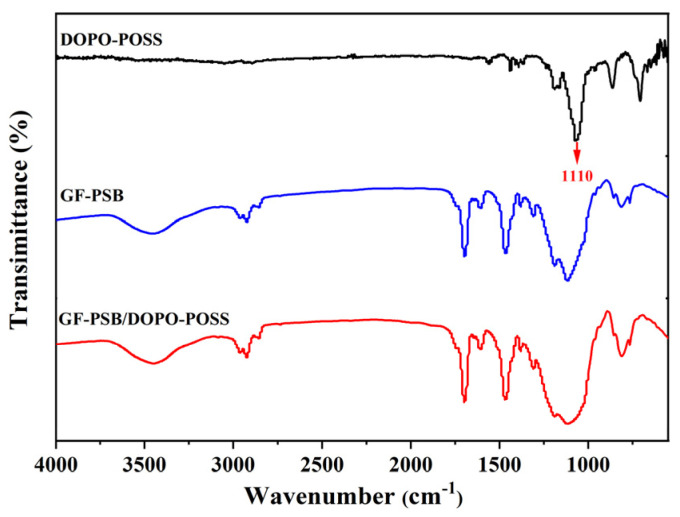
IR spectra of the samples of DOPO-POSS, GF-PSB, and GF-PSB/DOPO-POSS (the content of DOPO-POSS is 10 wt.%).

GF, PSB, and DOPO-POSS were selected as the reinforced fiber, polymer resin, and flame retardant, respectively, for the preparation of the insulated composite laminate. Figure 3 shows the IR spectra of representative samples. The IR spectrum of GF-PSB exhibits several adsorption peaks including 3451, 2961, 2925, 2857, 1698, 1604, 1467, 1192, 857, 813, and 766 cm^−1^, which cover almost all the weak adsorption peaks of DOPO-POSS. However, the GF-PSB/DOPO-POSS samples exhibit a broader and stronger peak than GF-PSB does in the range of 1000 to 1200 cm^−1^, which clearly indicating the presence of DOPO-POSS. The strong characteristic adsorption of DOPO-POSS at 1110 cm^−1^ (the Si-O bond) should have enhanced the composites’ adsorption in this region.

### 3.2. Dielectric Properties

**Figure 4:**
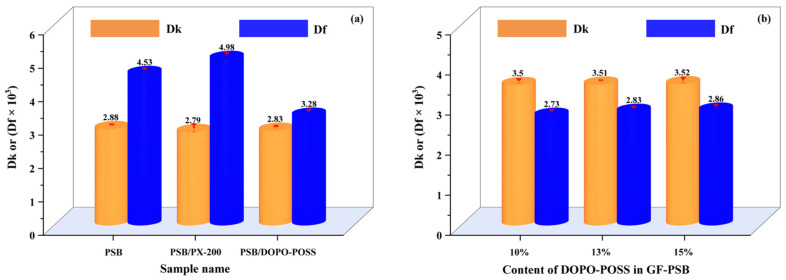
(**a**) Dk and Df values of PSB composites at 10 GHz (without GF, the thickness of sample is 0.09 mm, the contents of PX-200 in PSB/PX-200 and DOPO-POSS in PSB/DOPO-POSS are 20 wt.%). (**b**) Dk and Df values of the GF-PSB composite laminates with the different DOPO-POSS contents (with GF, the thickness is 0.25 mm).

Insulated composite laminate with low Dk and Df is essential for the preparation of high-speed and high-frequency CCL, which is conducive to improving the signal transmission speed and reducing the heat release. As shown in Figure 4a, the Df of PSB/DOPO-POSS composite (0.09 mm in thickness, without GF) is 0.00328, which is significantly lower than PSB with PX-200 at the same thickness (Df: 0.00498). The data suggest that the dielectric properties of DOPO-POSS are significantly better than those of PX-200, which is one of the most commonly used commercial flame retardants in high-speed and high-frequency CCL fields. It is important to note that the addition of most flame retardants can lead to an increase in Df and a deterioration of material properties. However, the addition of DOPO-POSS in 20 wt.% actually lowers the laminate’s Df from 0.00453 to 0.00328, which could be attributed to the unique structure of POSS with a low polar Si-O-Si skeleton and cage nanostructure [29,41]. To provide a more comprehensive evaluation of the DOPO-POSS performance, the Dk values of different laminates were also examined. As exhibited in Figure 4a, the addition of 20 wt.% of DOPO-POSS does not increase the Dk of PSB composite, just like PX-200. These results demonstrate that DOPO-POSS has a promising application potential in the CCL field. Figure 4b shows the Dk and Df values of GF-reinforced PSB composite laminates (0.25 mm of thickness) with different DOPO-POSS contents. The Dk values remain almost constant as the increase of DOPO-POSS content increases from 10% to 15%, while the Df values increase from 0.00273 to 0.00286 with the increase of DOPO-POSS content, which exhibits a similar effect as SiO_2_ filler [43]. Therefore, an appropriate amount of content could decrease the Df (Figure 4a), but higher content would lead to an increase of Df (Figure 4b). Considering Df is a key factor that affects the attenuation of signal propagation [44], GF-PSB with 10 wt.% DOPO-POSS content was adopted for other composite laminates’ preparation and related tests.

### 3.3. Thermal Properties and Dynamic Mechanical Analysis

**Figure 5:**
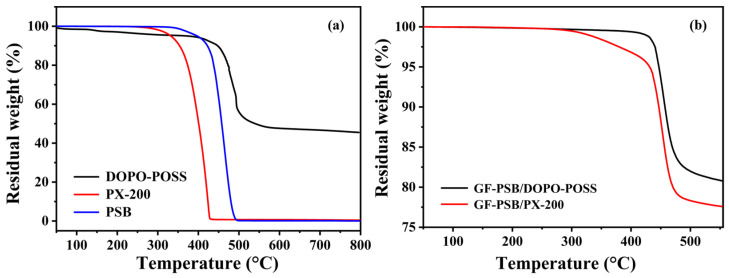
(**a**) TG curves of DOPO-POSS, PX-200, and PSB. (**b**) TG curves of flame-retardant GF-PSB composites (the contents of PX-200 in PSB/PX-200 and DOPO-POSS in PSB/DOPO-POSS are 10 wt.%).

The thermal degradation behaviors of PSB, DOPO-POSS, PX-200, and flame-retardant GF-PSB composites in a N_2_ atmosphere are shown in Figure 5, and the corresponding data are presented in Table S1. It is obvious that all samples show a one-step decomposition process; PSB and PX-200 exhibit similar thermal degradation characteristics. The *T*_5%_ and *T*_max_ values for PSB are 397 °C and 460 °C, respectively, with a final char residue of almost 0. The *T*_5%_ and *T*_max_ of PX-200 are 332 °C and 401 °C, respectively, and it almost completely decomposes at 430 °C. For DOPO-POSS, its *T*_5%_ and *T*_max_ are 373 °C and 493 °C, respectively, which is significantly higher than that of PX-200. Furthermore, the final char residue of DOPO-POSS is 48.9%, which could be attributed to the POSS structure. These results suggest that the thermal stability of DOPO-POSS is superior to PX-200 and PSB. Figure 5b shows the TG curves of GF-PSB with DOPO-POSS or PX-200 as flame retardants. The *T*_5%_ of GF-PSB/DOPO-POSS is 446 °C, which is higher than that of GF-PSB/PX-200 (431 °C). In addition, the residue of GF-PSB/DOPO-POSS is 80.8%, also higher than that of GF-PSB/PX-200 (77.0%), which could be attributed to the excellent char-forming ability of DOPO-POSS. As a result, DOPO-POSS has great potential for use in manufacturing CCLs due to its high initial decomposition temperature, which improves processability and long-term stability in high-temperature environments [45].

**Figure 6:**
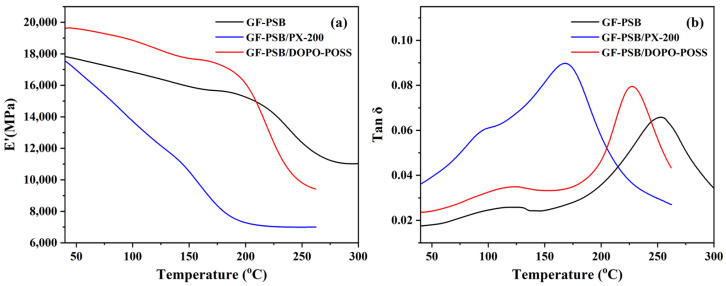
(**a**) Dynamic storage modulus and (**b**) tan δ of various laminates at 1 Hz (the contents of PX-200 in PSB/PX-200 and DOPO-POSS in PSB/DOPO-POSS are 10 wt.%).

DMA was also used to evaluate the performance of the laminates. The storage modulus value (E′) and loss factor (tan δ) are shown in Figure 6a,b, respectively, and the corresponding data are listed in Table S2. As shown in Figure 6a, the E’ of GF-PSB/DOPO-POSS before the glass transition temperature (*T*_g_) is larger than that of GF-PSB and GF-PSB/PX-200, demonstrating that DOPO-POSS has a reinforcing effect on the dynamic modulus. These could be attributed to the nano-scale dispersion of the DOPO-POSS additive in the PSB matrix [36]. However, the E’ of GF-PSB/DOPO-POSS above the 209 °C is smaller than that of GF-PSB, which may be caused by the relatively low *T*_g_ (131.18 °C) of DOPO-POSS [41]. As shown in Figure 6b and Table S2, the *T*_g_ of GF-PSB/DOPO-POSS is lower than that of GF-PSB. The result may be attributed to the increased free volume of the PSB-rich phase, in which some DOPO-POSS molecules gain access to the void space of the PSB structure. Another reason may be that the DOPO-POSS could partially dissolve in the PSB matrix upon heating.

### 3.4. Flame-Retardant Properties

To evaluate the flame-retardant properties of GF-PSB/DOPO-POSS, LOI and UL-94 tests were conducted; the results are listed in Table 2. It is obvious that with the addition of DOPO-POSS, the LOI increases from 47.7 to 51.1. And, the LOI of GF-PSB/DOPO-POSS (51.1) is higher than GF-PSB/PX-200 (48.4), suggesting that the flame-retardant effect of DOPO-POSS is better than PX-200.

The UL-94 rating is another parameter to evaluate flame-retardant properties. As exhibited in Table 2, the UL-94 rating of GF-PSB/DOPO-POSS is V-1, which is similar to that of GF-PSB/PX-200, while the UL-94 rating of GF-PSB is only V-2. Additionally, GF-PSB exhibited obvious dripping phenomenon during the combustion process, while the dripping totally disappeared with the addition of PX-200 or DOPO-POSS. Furthermore, the inclusion of DOPO-POSS has significantly reduced the *t*_1_ + *t*_2_ value from 182 s to 79 s. The reduction is even greater than that of GF-PSB/PX-200 (85 s), indicating the clear flame-retardant effect of DOPO-POSS.

**Figure 7:**
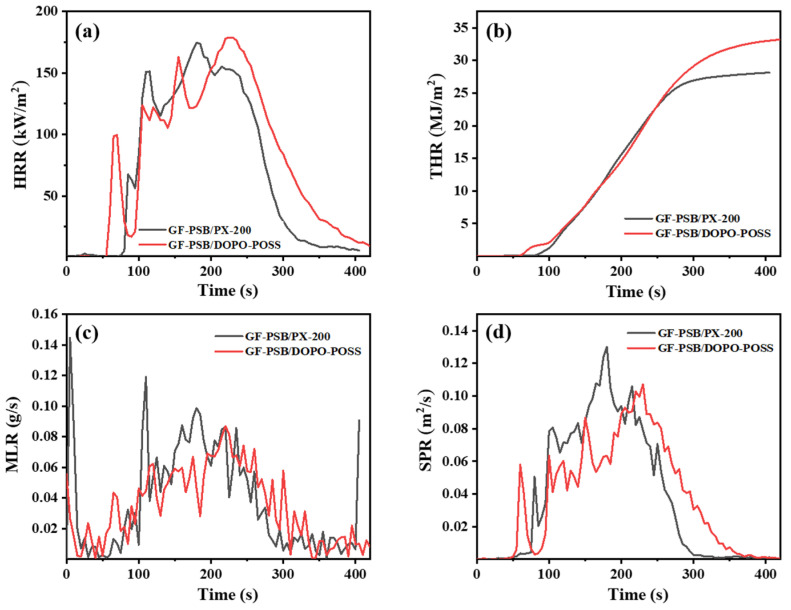
(**a**) HRR, (**b**) THR, (**c**) MLR, and (**d**) SPR curves of flame-retarded GF-PSB composites (the contents of PX-200 in PSB/PX-200 and DOPO-POSS in PSB/DOPO-POSS are 10 wt.%).

To further estimate the flame-retardant effects of DOPO-POSS in GF-PSB/DOPO-POSS, the combustion performance of laminates was examined through a cone calorimeter test, and the digital pictures of composites after the test are shown in Figure S1. The heat release rate (HRR), total heat release (THR), mass loss rate (MLR), and smoke production rate (SPR) curves are presented in Figure 7a–d, respectively, and the relevant data are summarized in Table S3. The average heat release rate (av-HRR), THR, and average effective heat of combustion (av-EHC) of GF-PSB/DOPO-POSS composites are slightly higher than those of GF-PSB/PX-200. 

According to Figure 7a, both GF-PSB/DOPO-POSS and GF-PSB/PX-200 show four peaks in the HRR curves. The ignition time (TTI) of GF-PSB/DOPO-POSS is 54 s, and the heat is transferred from the surface to the interior. The peak heat release rate (PHRR) is 179 kW/m^2^, which appears in 225 s, and the flameout time (TTF) is 320 s. In Figure 7b, the THR curves of both laminates show a similar trend of rising rapidly at first and then rising gradually. The total heat release of the GF-PSB/DOPO-POSS curve is larger, and the inflection point appears at about 320 s. Combined with Figure 7c, the peak value of the MLR curve of GF-PSB/DOPO-POSS is lower, which reduces the decomposition rate of the PSB matrix to a certain extent, and the curve plateaus at about 340 s with the formation of the carbon layer. Figure 7d is the smoke-production rate. In actual fire conditions, smoke is the main cause of death, so the lower release of smoke is a very important factor of flame-retardant materials. The average specific extinction area (av-SEA) and average CO yield (COY) of GF-PSB/DOPO-POSS laminate are 1168.881 m^2^/kg and 0.1132 kg/kg (Table S3), lower than those of GF-PSB/PX-200 laminate, whereas the total smoke production (TSP) and average CO_2_ yield (CO_2_Y) are slightly higher than the latter. The reason may be twofold. Firstly, before the flame retardant is effective, the PSB matrix burns and releases smoke containing small flammable organic compounds. Secondly, both PSB and DOPO-POSS contain many phenyl groups, and the aromatic ring may increase the amount of flue gas released during pyrolysis.

**Figure 8:**
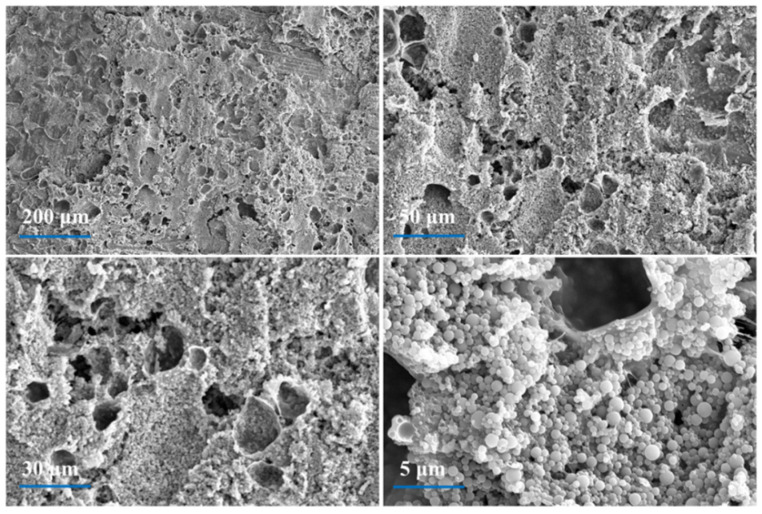
SEM micrographs of the top layer of GF-PSB/DOPO-POSS laminate after cone calorimeter test (the thickness of laminate is 0.25 mm, the content of DOPO-POSS is 10 wt.%). The four images come from the same location with different magnifications.

After a cone calorimeter test, the top layer of GF-PSB/DOPO-POSS laminate was taken, and the SEM images of char residues on the surface are exhibited in Figure 8. It is clear that, except for the dense residual carbon region, a discontinuous structure and some tiny holes on the char surface of GF-PSB/DOPO-POSS can be observed. The result indicates that during the combustion process, combustible volatiles and non-combustible gas were released from the internal matrix of the GF-PSB/DOPO-POSS system. The gas flow and heat flux should have caused the broken bubble structures. Moreover, it is likely that the DOPO structure decomposed into oxygen-containing phosphoric acid, accelerating the dehydration and carbonization of the system. Simultaneously, the POSS structure decomposed into SiO_2_ covering the carbon layer [40]. In addition, some spherical inorganic fillers also can be seen. These components together formed the condensed phase. It is reasonable to assume that the flame-retardant mechanism of DOPO-POSS in GF-PSB composites consists of both gas-phase and condensed-phase mechanisms.

## 4. Conclusions

In summary, DOPO-POSS, synthesized using a recently improved method, has been applied in the flame-retardant modification of the PSB matrix and for the fabrication of high-performance composite laminate with GF. The GF-PSB/DOPO-POSS composite laminate, 0.25 mm in thickness with 10 wt.% DOPO-POSS content, exhibited low Dk (3.5) and low Df (0.00273), which could meet the ultra-low dielectric standard. Furthermore, compared with GF-PSB, with PX-200 as the flame retardant, GF-PSB/DOPO-POSS laminates show superior thermal and dynamic mechanical properties. The flame-retardant performance of GF-PSB/DOPO-POSS reached a UL-94V-1 rating, and did not drip during the burning process. More importantly, the LOI and self-extinguishing times of GF-PSB/DOPO-POSS are 51.1% and 79 s, respectively, exhibiting better flame retardancy performance than PX-200. The cone calorimeter test revealed that the heat release and total smoke generation of GF-PSB/DOPO-POSS composite were both slightly higher than GF-PSB/PX-200 composite. However, the average CO yield (COY) of GF-PSB/DOPO-POSS laminate was lower than that of the GF-PSB/PX-200 laminate, indicating that modification with DOPO-POSS could reduce the toxicity of the released gases. Taken together, DOPO-POSS has great potential in the fabrication of flame-retardant PCBs for high-frequency and high-speed signal propagation.

## Figures and Tables

**Table 1:** Formulations of various composites and composite laminates.

Samples	Matrix Composition (wt.%)				
	PSB	PX-200	DOPO-POSS	GF	Other Additives ^1^
PSB	83	0	0	0	17
PSB/PX-200	63	20	0	0	17
PSB/DOPO-POSS	63	0	20	0	17
GF-PSB/DOPO-POSS-10	40	0	10	33	17
GF-PSB/DOPO-POSS-13	37	0	13	33	17
GF-PSB/DOPO-POSS-15	35	0	15	33	17

^1^ Curing agent and SiO_2_ fillers.

**Table 2:** LOI values and UL-94 ratings of GF-PSB and flame-retarded GF-PSB composites ^1^.

Samples	LOI (%)	UL-94	(*t*_1_ + *t*_2_) (s)	Dripping
GF-PSB	47.7 ± 0.1	V-2	182	Yes
GF-PSB/PX-200 ^2^	48.4 ± 0.2	V-1	85	No
GF-PSB/DOPO-POSS ^3^	51.1 ± 0.1	V-1	79	No

^1^ Thickness of composite is 0.25 mm. ^2^ Content of PX-200 is 10 wt.%. ^3^ Content of DOPO-POSS is 10 wt.%.

## Data Availability

The data presented in this study are available on request from the corresponding author.

## Supplementary Materials

The following supporting information can be downloaded at https://www.mdpi.com/article/10.3390/polym16040544/s1. Figure S1: Digital pictures of the residue of (a) GF-PSB/PX-200 and (b) GF-PSB/DOPO-POSS composites after cone calorimeter tests; Table S1: TGA data of the samples; TableS2: DMA results for various laminates; Table S3: cone calorimeter data for the flame-retarded PSB.
