# Supplementary material for: Flame-Retardant GF-PSB/DOPO-POSS Composite with Low Dk/Df and High Thermal Stability for High-Frequency Copper Clad Applications
Source: Polymers (Basel). 2024 Feb 17;16(4):544. doi: 10.3390/polym16040544 (PMC10892954; doi:10.3390/polym16040544)
Supplement: Supplementary file 1 [file polymers-16-00544-s001.zip › polymers-2836930-supplementary.pdf]

Supporting Information

Flame-Retardant GF-PSB/DOPO-POSS Composite Laminate with Low Dk/Df and High Thermal Stability for High-Frequency Copper Clad Laminate

Ke Zheng [1], Yizhi Zhang [1], Jiaxiang Qiu [2], Guanqun Xie [2,*], Zengbiao Huang [3], Wei Lin [3], Zhimeng Liu [1], Qianfa Liu [3], Xiaoxia Wang [1,*]

[1] Subcenter of Dongguan University of Technology of National Engineering Research Center of Electronic Circuits Base Materials, School of Materials Science and Engineering, Dongguan University of Technology, Dongguan 523808, China; zhengke@dgut.edu.cn (K.Z.); 1150777934@qq.com (Y.Z.); 806953921@qq.com (Z.L.)

[2] School of Environment and Civil Engineering, Dongguan University of Technology, Dongguan, 523808, PR China; 915849945@qq.com (J.Q.);

[3] National Engineering Research Center of Electronic Circuits Base, Materials SHENGYI Technology Co. Ltd., Dongguan 523808, China; huangzb@syst.com.cm (Z.H.); linw@syst.com.cn (W.L.); Liuqf@syst.com.cn (Q.L.)

[*] Correspondence: wangxx@dgut.edu.cn (X.W.); gqxie@dgut.edu.cn (G.X.)

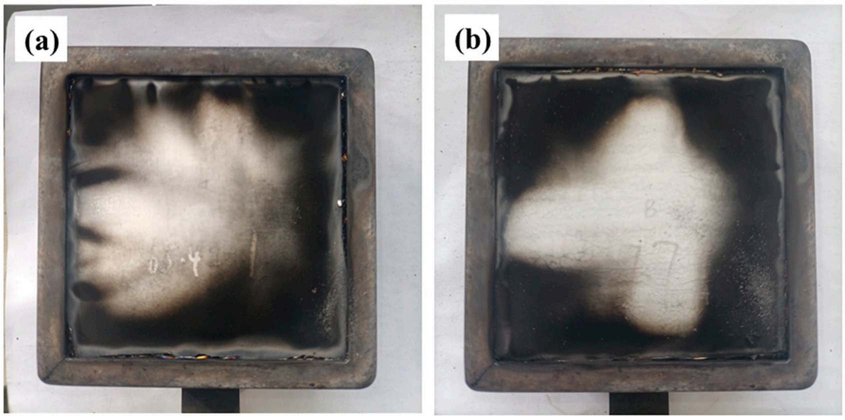

Figure S1. Digital pictures of the residue of (a) GF-PSB/PX-200 and (b) GF-PSB/DOPO-POSS composites after cone calorimeter tests.

Table S1. TGA data of the samples.

Samples	$T_{5\%}$ (°C) [a]	T_{max} (°C) [b]	Residues (%, 555 °C)
PSB	397	460	0.5
PX-200	332	401	0.7
DOPO-POSS	373	493	48.9
GF-PSB/PX-200	431	450	77.0
GF-PSB/DOPO-POSS	446	461	80.8

[a] $T_{5\%}$ is the temperature with 5% weight loss. [b] T_{max} is the temperature with maximum decomposition rate.

Table S2. DMA results for various laminates.

Samples	E' at 50 °C (MPa)	E' at 150 °C (MPa)	E' at 250 °C (MPa)	T_g (°C)
GF-PSB	17677	15923	12365	253
GF-PSB/PX-200	16971	10520	6998	168
GF-PSB/DOPO-POSS	19600	17709	9816	227

Table S3. Cone calorimeter data for the flame-retarded PSB.

Composites	GF-PSB/PX-200	GF-PSB/DOPO-POSS
av-HRR (kW/m^2)	86.30	88.76
THR (MJ/m^2)	28	33
TSP (m^2)	15.355	15.489
av-COY (kg/kg)	0.1435	0.1132
av-CO_2Y (kg/kg)	1.1847	1.4087
av-EHC (MJ/kg)	19.226	22.23
av-MLR (g/s)	0.0398	0.035
av-SEA (m^2/kg)	1180.014	1168.881
